# Cyclooxygenase-2 Is a Target of MicroRNA-16 in Human Hepatoma Cells

**DOI:** 10.1371/journal.pone.0050935

**Published:** 2012-11-30

**Authors:** Noelia Agra Andrieu, Omar Motiño, Rafael Mayoral, Cristina Llorente Izquierdo, Ana Fernández-Alvarez, Lisardo Boscá, Marta Casado, Paloma Martín-Sanz

**Affiliations:** 1 Instituto de Investigaciones Biomédicas Alberto Sols, (Centro Mixto Consejo Superior de Investigaciones Científicas-Universidad Autónoma de Madrid, CSIC-UAM), Madrid, Spain; 2 Centro de Investigación Biomédica en Red de Enfermedades Hepáticas y Digestivas (CIBERehd), Barcelona, Spain; 3 Instituto de Biomedicina de Valencia del Consejo Superior de Investigaciones Científicas (IBV-CSIC), Valencia, Spain; Centro de Investigación en Medicina Aplicada (CIMA), Spain

## Abstract

Cyclooxygenase-2 (COX-2) expression has been detected in human hepatoma cell lines and in human hepatocellular carcinoma (HCC); however, the contribution of COX-2 to the development of HCC remains controversial. COX-2 expression is higher in the non-tumoral tissue and inversely correlates with the differentiation grade of the tumor. COX-2 expression depends on the interplay between different cellular pathways involving both transcriptional and post-transcriptional regulation. The aim of this work was to assess whether COX-2 could be regulated by microRNAs in human hepatoma cell lines and in human HCC specimens since these molecules contribute to the regulation of genes implicated in cell growth and differentiation. Our results show that miR-16 silences COX-2 expression in hepatoma cells by two mechanisms: a) by binding directly to the microRNA response element (MRE) in the COX-2 3′-UTR promoting translational suppression of COX-2 mRNA; b) by decreasing the levels of the RNA-binding protein Human Antigen R (HuR). Furthermore, ectopic expression of miR-16 inhibits cell proliferation, promotes cell apoptosis and suppresses the ability of hepatoma cells to develop tumors in nude mice, partially through targeting COX-2. Moreover a reduced miR-16 expression tends to correlate to high levels of COX-2 protein in liver from patients affected by HCC. Our data show an important role for miR-16 as a post-transcriptional regulator of COX-2 in HCC and suggest the potential therapeutic application of miR-16 in those HCC with a high COX-2 expression.

## Introduction

Hepatocellular carcinoma (HCC) is the fifth most common cancer worldwide and has an increasing incidence in western countries [1]. Although the risk factors for HCC are well characterized, the molecular pathogenesis of this tumor type is not well understood [2], [3], and thus the identification of new possible targets for the development of non-conventional treatments is urgent and must be improved.

Cyclooxygenase-1 (COX-1) and -2 catalyze the first step in prostanoid biosynthesis. COX-1 is constitutively expressed in many tissues, whereas COX-2 is induced by a variety of stimuli such as growth factors, pro-inflammatory stimuli, hormones and other cellular stresses [4]. Adult hepatocytes fail to induce COX-2 expression regardless of the pro-inflammatory factors used [5], [6]. However, our group and others demonstrated that partial hepatectomy (PH) [7], [8] induced COX-2 in hepatocytes and contributed to the progression of cell cycle after PH. In addition to liver regeneration after PH or hepatotoxic agents, expression of COX-2 has been detected in animal models of cirrhosis [9], in human hepatoma cell lines [10], [11], in human HCC [12] and after HBV and HCV infection [13], [14].

COX-2 is widely regarded as a potential pharmacological target for preventing and treating inflammatory and cancer diseases. Therapeutic strategies have focused primarily on selective inhibitors of COX-2 activity; however, considerable less attention has been paid to identifying anticancer agents that suppress the expression of COX-2 [15]. COX-2 overexpression is the result of the activation of many intracellular pathways that regulate COX-2 both at transcriptional and post-transcriptional level. The 5′-UTR of the COX-2 gene contains binding sites for numerous regulatory transcription factors including two NF-κB (nuclear factor κB) motifs, two AP-1 (activator protein 1) sites and two CREs (cAMP-response elements) among others [16]. However, the regulation of the expression of COX-2 is more complex including modifications of genomic DNA and chromatin and at the post-transcriptional level via targeting its 3′-UTR [17]. The 3′-UTR of COX-2 contains multiple copies of AU-rich elements (AREs) and microRNA response element (MRE) motifs which, when bound by specific ARE-binding factors or miRNAs, influence COX-2 stability and translational efficiency [17].

MicroRNAs (miRNAs) are short single-stranded non-coding RNAs that influence post-transcriptional gene regulation by affecting mRNA stability and/or translational repression of their target mRNAs [18]. Alterations of the expression pattern of miRNAs that regulate genes involved in cellular proliferation, differentiation or apoptosis, have been found in different human tumors including HCC [19], [20], suggesting that they may represent a novel class of oncogenes or tumor suppressor genes. Moreover, recent reports of profound phenotypic abnormalities in miRNA-knockout models further demonstrate their crucial roles as regulators of gene expression [21]. Regarding COX-2, Dey’s group [22], [23] highlighted a miRNA-mediated regulation of COX-2 by mmu-miR-101a and mmu-miR-199a* during embryo implantation and in endometrial cancer cells. Recent works have reported that miR-101 downregulation is involved in COX-2 overexpression in human colon cancer cells (CRC) [24], miRNA-26b regulates the expression of COX-2 in desferrioxamine-treated carcinoma of nasopharyngeal epithelial cells [25] and binding of miR-16 to AREs of TNF-α, IL-6, IL-9 and COX-2 mRNA transcripts could promote their degradation [20], [26].

Besides miRNAs, various cytoplasmic proteins have been reported to bind the COX-2 3′UTR [20]. As an example, the RNA-binding protein CUGBP2 interacts directly with specific AREs within the first 60 nucleotides of the COX-2 3′-UTR and that binding stabilizes the COX-2 mRNA yet inhibits its translation [27]; tristetrapolin binds to COX-2 3′UTR and decrease mRNA levels in colon cancer [28], whereas Human Antigen R (HuR) is a translational enhancer of COX-2 in ovarian carcinoma [29] and in colon carcinogenesis [20].

To our knowledge, no data is available concerning the post-transcriptional regulation of COX-2 by miRNAs and RNA-binding proteins in HCC. Our results show that miR-16 silences COX-2 expression in hepatoma cells by two mechanisms: by binding directly to the MRE motif in the COX-2 3′-UTR and by decreasing the levels of HuR. miRNA-16 is able to inhibit cell proliferation, to promote cell apoptosis and to suppress the ability of WRL68 hepatoma cell line to develop tumors in nude mice partially through targeting COX-2 expression. Moreover a reduced miR-16 expression tends to correlate to high levels of COX-2 protein in liver from patients affected by HCC. Our data suggest an important role for miR-16 in HCC and implicate the potential therapeutic application of miR-16 in those HCC with a high COX-2 expression.

**Figure 1:**
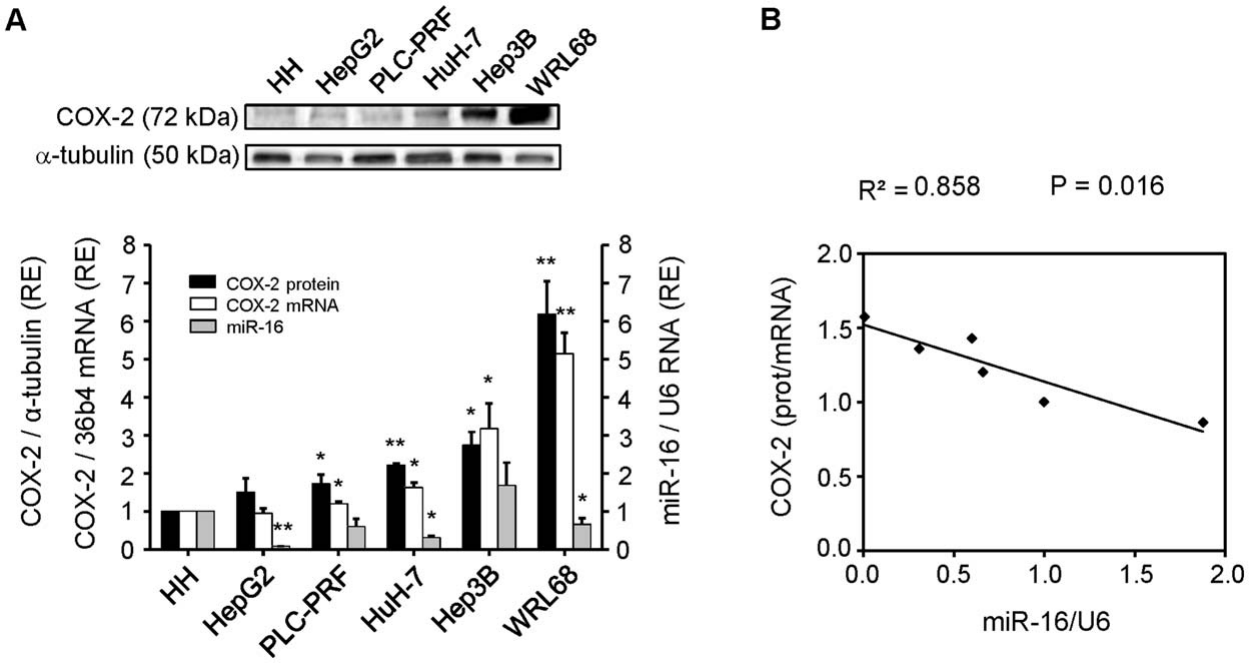
miR-16 and COX-2 correlate inversely in HCC cell lines. Cells were plated in 100-mm dishes and grown to 60–70% confluence in culture medium supplemented with 10% FBS. (**A**) Total cellular extracts were prepared from HCC cells and protein (30–50 µg/lane) was analyzed by Western blot. A representative Western blot showing COX-2 protein. The expression of target protein was normalized to that of α-tubulin. Densitometric analysis of COX-2 expression (black bars) is referring to HH as 1 and expressed as relative expression (RE). Total RNA was prepared from HCC cell lines and COX-2 mRNA was analyzed by real-time PCR. COX-2 mRNA amounts (white bars), normalized to the expression of 36b4 mRNA, and miR-16 expression (grey bars), normalized against U6 RNA levels, were calculated. Values represent fold change relative to human hepatocytes (HH) as 1. Data are reported as means±SD of three independent experiments. **p<0.01 and *p< 0.05 vs. the HH. (**B**) The inverse correlation between COX-2 protein/mRNA ratio and miR-16 expression in HCC cells is graphically depicted. The coefficient of determination (R^2^) was calculated.

**Table 1:** Expression of selected miRNAs is evaluated in HCC cell lines.

Cell Lines			
	miR-16 (R^2^ = 0.86)	miR-26b (R^2^ = 0.43)	miR-101 (R^2^ = 0.71)
	*p = 0.016*	*p = 0.297*	*p = 0.018*
**HH**	1	1	1
**HepG2**	0.008±0.002	0.023±0.004	0.141±0.003
**HuH-7**	0.309±0.150	0.114±0.100	0.573±0.250
**PLC-PRF**	0.601±0.300	0.021.±0.015	0.383±0.800
**WRL68**	0,661±0.234	0.019±0.009	0.451±0.315
**Hep3B**	1.578±0.015	0.682±0.261	1.956±0.394
	**miR-199a (R^2^ = 0.49)**	**miR-122 (R^2^ = 0.03)**	**miR-21 (R^2^ = 0.02)**
	***p = 0.226***	***p = 0.241***	***p = 0.919***
**HH**	1	1	1
**HepG2**	0.583±0.056	0.001±7.65E-05	1.501±0.121
**HuH-7**	1.084±0.430	0.028±0.005	1.558±0.313
**PLC-PRF**	0.702±0.359	0.004±1.91E-05	1.130±0.600
**WRL68**	0.479±0.171	0.001±6.32E-05	0.997±0.006
**Hep3B**	2.321±0.380	0.012±0.003	1.741±0.154

The miRNAs (miR-16, miR-26b, miR-101, miR-199a, miR-122 and miR-21) were selected by using miRWalk computational analysis as described in Methods. The expression profile was analyzed in HCC cell lines using real-time PCR, normalized against U6 RNA levels and refers to human hepatocytes (HH) as 1. COX-2 protein/mRNA ratio was compared to miRNAs expression in HCC cells and the coefficient of determination (R^2^) was calculated.

## Materials and Methods

### Chemicals

Antibodies were from Santa Cruz Laboratories (Santa Cruz, CA, USA), Sigma Chemical Co. (St. Louis, MO, USA), Cell Signaling (Boston, MA, USA), Abcam (Cambridge, UK) and Cayman Chemical (Ann Arbor, MI, USA). Prostaglandin E_2_ (PGE_2_) was from Cayman Chemical. Reagents were from Roche Diagnostics (Mannheim, Germany) or Sigma Chemical Co. Reagents for electrophoresis were obtained from Bio-Rad (Hercules, CA, USA). Tissue culture dishes were from Falcon (Becton Dickinson Labware, Franklin Lakes, NJ, USA). Tissue culture media were from Gibco (Invitrogen™, Grand Island, NY, USA).

**Figure 2:**
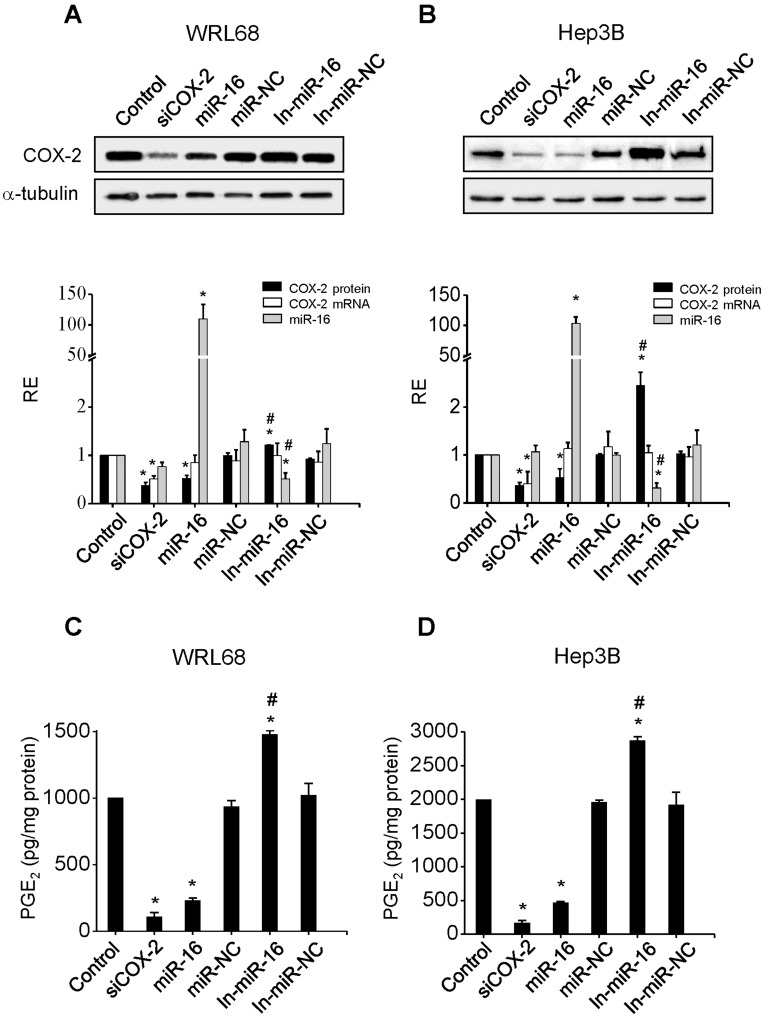
miR-16 regulates COX-2 expression in HCC cell lines. WRL68 and Hep3B cells were transfected with: 30 nM siRNA anti-COX2 (siCOX-2) or 50 nM of miR-16, miR-16 inhibitor (In-miR-16), miR negative control (miR-NC) or miR negative control inhibitor (In-miR-NC). (**A–B**) COX-2 protein was analyzed by Western blot 48 h after transfection and normalized against α-tubulin protein. COX-2 mRNA and miR-16 expression were analyzed by real-time PCR. COX-2 mRNA and miR-16 expression were normalized against 36b4 mRNA and U6 RNA levels, respectively. Relative expression of each sample refers to control as 1 (cells transfected only with lipofectamine). (**C–D**) PGE_2_ concentration was determined by enzyme immunoassay in the supernatant of the cells. Data are reported as means±SD of four independent experiments. *p< 0.05 vs. the control condition and # *p< 0.05 vs*. the miR-16 transfection condition.

### Patients

Seven individual tumoral and paired non-tumoral HCC human samples were obtained from de Spanish Tumor Bank Network of the Centro Nacional de Investigaciones Oncológicas (CNIO). Institutional review board approval (N°PI. CEI PI 20_2011) was obtained for these studies from Comité de la Investigación y de Bienestar Animal of CNIO and all participants provided written informed consent. Tissues were evaluated by pathologists by means of hematoxylin/eosin staining. Tissue was snap-frozen in liquid nitrogen and total RNA and protein were isolated as described below.

**Figure 3:**
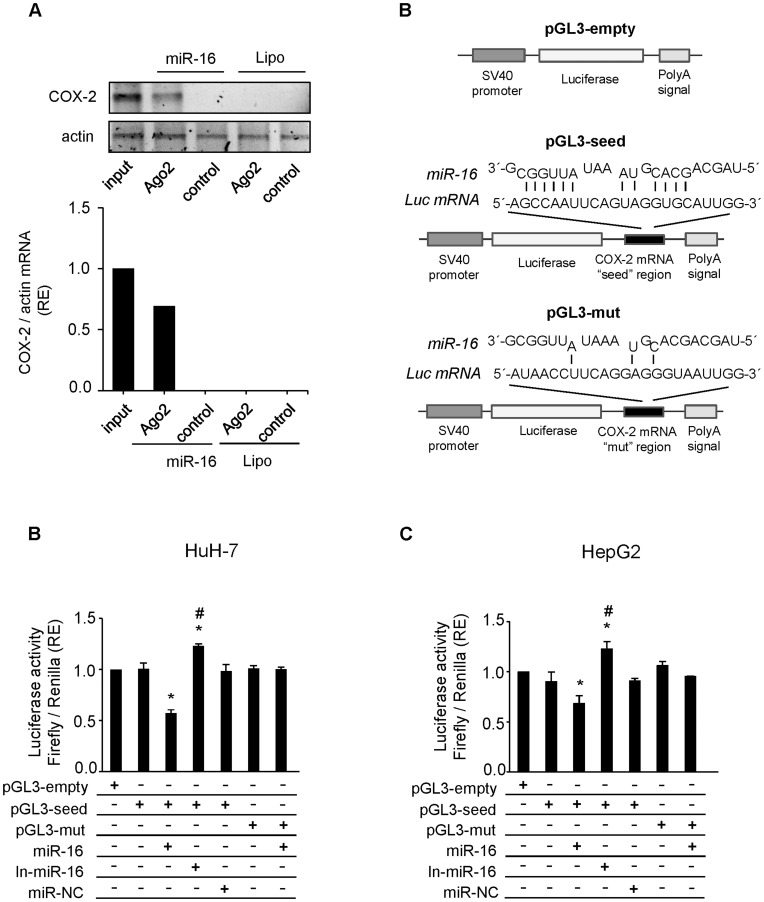
miR-16 binds COX-2 mRNA and inhibits its translation. (**A**) WRL68 cell extracts (500 µg per lane) were immunoprecipitated with Ago-2 or IgG antibodies. Bound RNA was harvested with TRIzol reagent, reverse transcriptased, and PCR amplified with COX-2 primers. PCR products were visualized by electrophoresis in SYBR Safe DNA gel stain agarose gels. The presence of COX-2 mRNA in WRL68 cell transfected with miR-16 or Lipofectamine after Ago2 immunoprecipitation was assessed, and fold differences were plotted. Input, total mRNA in cell extract; and control, bound mRNA after immunoprecipitation with IgG antiboby. (**B**) Scheme of pGL3-empty, pGL3-seed and pGL3-mut reporter vectors. In pGL3-seed, the putative binding site of miR-16 on COX-2 mRNA 3′-UTR region (as detected by RNAhybrid software) was introduced downstream luciferase gene. In pGL3-mut this region was mutated in order to avoid the binding between miR-16 and Luc mRNA. (**C–D**) A luciferase assay was carried out on HuH-7 and HepG2 cell lines using pGL3-seed and pGL3-mut reporter vectors. Firefly luciferase activity was evaluated 48 h after co-transfection with pGL3-empty/seed/mut (750 ng), miR-16 (50 nM), In-miR-16 (50 nM) and miR-NC (50 nM) as indicated. Data were normalized against renilla luciferase activity (all samples were co-transfected with 50 ng pRL vector and refer to the positive control, pGL3 empty vector). Data are reported as means±SD of three independent experiments. *p< 0.05 vs. the pGL3-empty condition and # *p< 0.05 vs*. the miR-16 transfection condition.

### Cell Culture

The cell lines WRL68, HepG2 and Hep3B were purchased from the American Type Culture Collection, ATCC (Manassas, VA, US). All these cells lines were authenticated by ATCC and were expanded twice, and stored in liquid N_2_. Expansions from these clones were used up to 6 months in culture. PLC/PRF/5 [30] was kindly provided by Dr. C Perret (Institut Cochin, CNRS UMR8104, University Paris-Descartes, Paris, France) and HuH-7 [31] by Dr. M. Kern (Department of General Pathology, University Hospital Heidelberg, Heidelberg, Germany). WRL68 was derived from human liver embryo. HepG2, Hep3B and HuH-7 are well differentiated hepatocellular carcinoma cell lines and PLC/PRF/5 is a malignant liver cancer with HBsAg positive cell line. Cells were grown on Falcon tissue culture dishes in EMEM or DMEM supplemented with 10% FBS and antibiotics (50 µg each of penicillin, streptomycin and gentamicin per ml) at 37°C in a humidified air 5% CO_2_ atmosphere. Human hepatocytes were from HPCH10 CryostaX™, Single-freeze Cryopreserved Pooled Human Hepatocytes (Xenotech, Lenexa, KA, USA).

### RNA Extraction and Quantitative Real-time PCR Analysis

Total RNA from HCC cells or human biopsies was extracted by using TRIzol reagent (Invitrogen, Grand Island, NY, USA). RNA (1 µg) was reverse transcribed using a Transcriptor First Strand cDNA Synthesis Kit following manufacturer’s indications (Roche Applied Science). For quantification of mature miRNAs, total RNA was extracted using the miRNeasy Mini Kit (Qiagen, Valencia, CA, USA). RNA (500 ng) was polyadenylated and reverse- transcribed to cDNA using the NCode™ miRNA first-strand cDNA synthesis kit (Invitrogen) in accordance with the manufactureŕs instructions. The cDNA was used as template for real-time PCR through Taqman probes. Primers for COX-2 (Hs00153133-m1) and 36b4 (Hs99999902-m1) were from Applied Biosystems (Carlsbad, CA, USA). Real-time PCR was performed using a MyiQ detection system (Bio-Rad) and thermocycling parameters were 95°C for 10 min, 50 cycles of 95°C for 15 s followed for 60°C for 1 min and finally 95°C for 1 min. Each sample was run in triplicate and was normalized to 36b4 mRNA. The replicates were then averaged, and fold induction was determined in a ΔΔCt based fold-change calculations.

**Figure 4:**
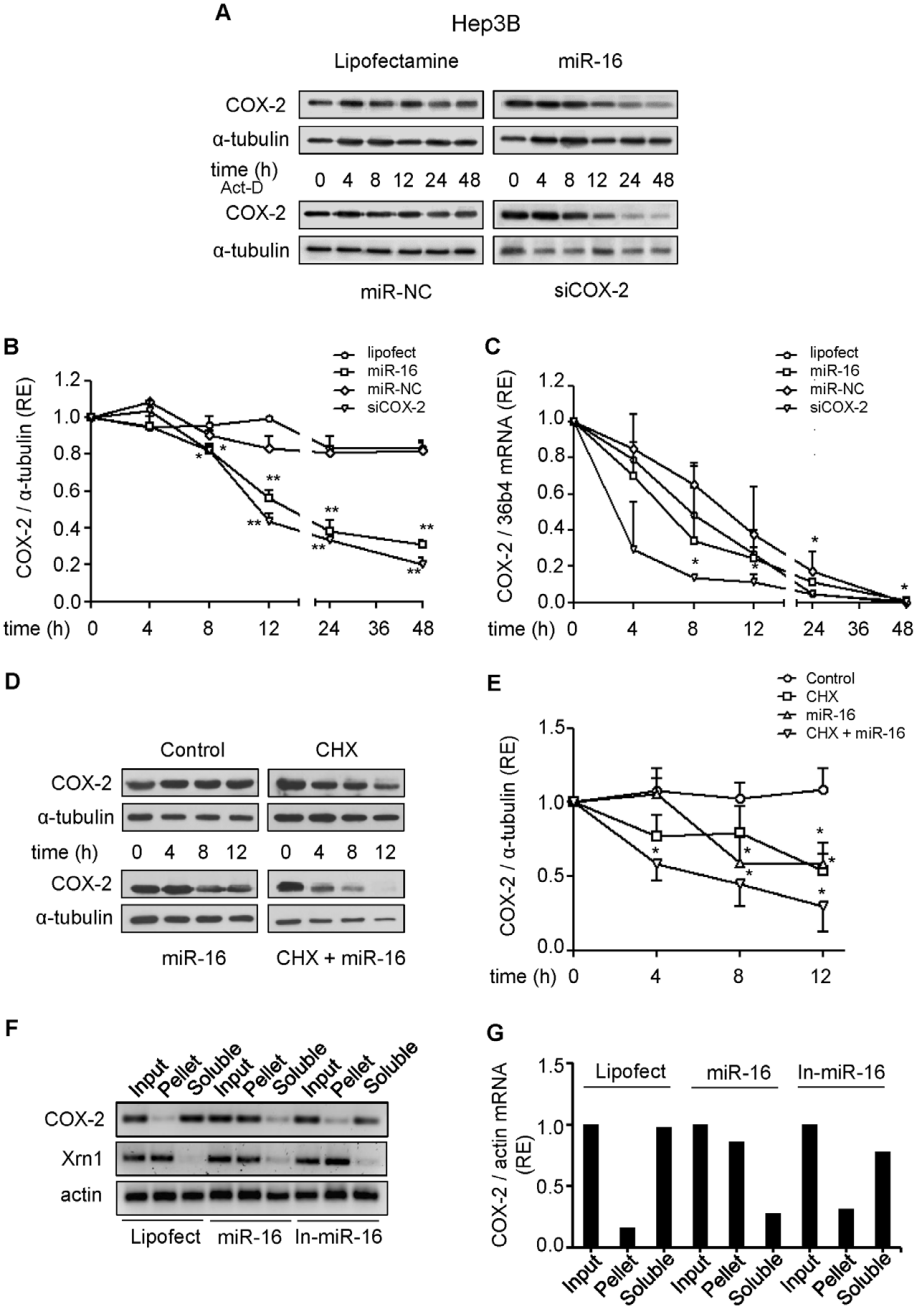
Effect of miR-16 on COX-2 mRNA and protein stability. Hep3B cells were transfected with 50 nM miR-16 or miR-NC, or 30 nM siCOX-2. 5 µg/ml actinomycin-D (Act D) or 10 µg/ml cycloheximide (CHX) were added after transfection. (**A–B**) COX-2 protein was analyzed by Western blot at different time after actinomycin-D treatment. Corresponding densitometry analysis is shown and the relative expression of each sample is related to sample at 0 h as 1. (**C**) mRNA COX-2 levels were analyzed by real time PCR. COX-2 mRNA amounts were calculated as relative expression and normalized to the expression of 36b4 mRNA. Values represent fold change relative to sample at 0 h. (**D–E**) COX-2 protein levels were analyzed by Western blot in the presence or absence of cycloheximide. Corresponding densitometric analysis is shown and the relative expression of each sample is related to the value at 0 h as 1. **F)** Hep3B cells were transfected with 50 nM miR-16, miR-16 inhibitor (In-miR-16) or lipofectamine and permeabilized with digitonine to obtain soluble and pellet fractions enriched in PB as described in Methods. RNA was isolated from each fraction with Trizol reagent, reverse transcriptased, and PCR amplified with COX-2, Xrn1 and actin primers. Input, RNA extracted from cells prior to fractionation. PCR products were visualized by electrophoresis in SYBR Safe DNA gel stain agarose gels. **G)** The presence of COX-2 mRNA in soluble and PB fractions was assessed and fold differences were plotted. Data are reported as means±SD of three independent experiments. **p<0.01 and *p<0.05 vs. the value of sample at 0 h.

Levels of miRNAs were quantified using the FastStart Universal SYBR Green Master (Roche) with the universal reverse primer provided in the kit and the following forward primers; hsa-miR-16: 5′- TAGCAGCACGTAAATATTGGCG -3′; hsa-miR-26b: 5′- CGCTTCAAGTAATTCAGGATAGGT -3′; hsa-miR-199a: 5′- CCCAGTGTTCAGACTACCTGTTC -3′; hsa-miR-101: 5′- CCGGTACAGTACTGTGATAACTGAA -3′; hsa-miR-21: 5′- CGGTAGCTTATCAGACTGATGTTGA -3′ and hsa-miR-122: 5′- TGGAGTGTGACAATGGTGTTT -3′. Thermocycling parameters were 95°C for 3 min and 40 cycles of 95°C for 15 s followed by 60°C for 30 sec. The expression of miRNAs was normalized against U6 snRNA levels (U6 primers: forward 5′- CTTCGGCAGCACATATACT -3′; reverse 5′- AAAATATGGAACGCTTCACG -3′). Melting curve analysis was performed to confirm the specificity of the PCR products.

**Figure 5:**
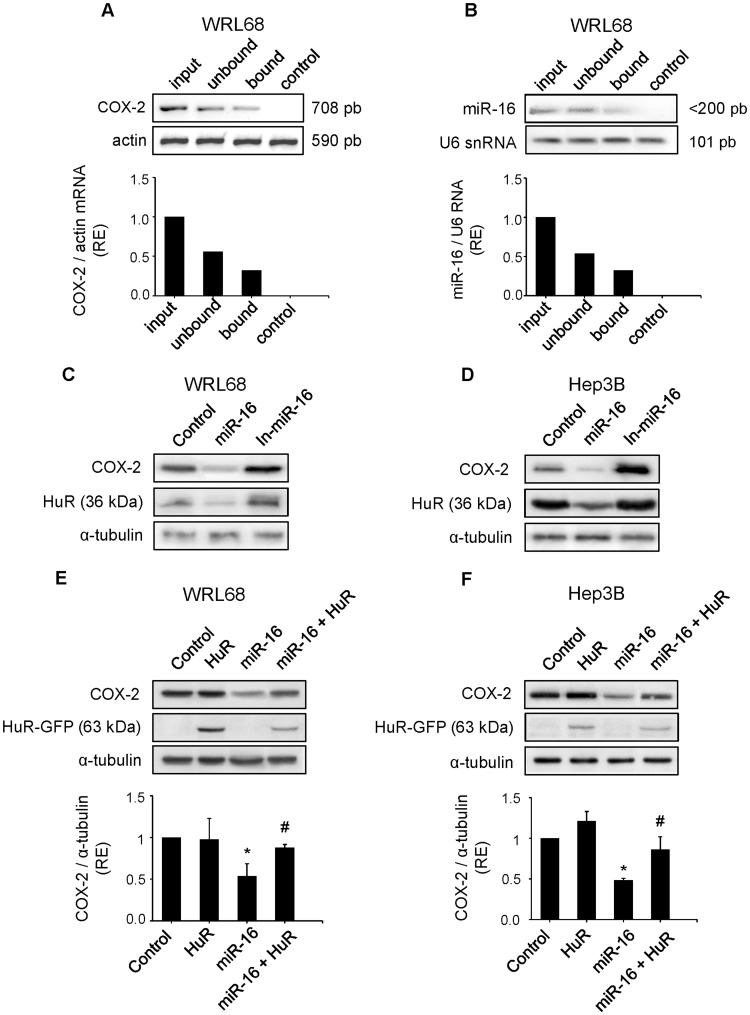
HuR antagonizes the downregulation of COX-2 expression caused by miR-16 in hepatoma cell lines. WRL68 cell extracts (500 µg per lane) were immunoprecipitated with HuR or IgG antibodies. Bound RNA was harvested with TRIzol reagent, reverse transcriptased, and PCR amplified with COX-2 (**A**) or miR-16 primers (**B**). PCR products were visualized by electrophoresis in SYBR Safe DNA gel stain agarose gels. The abundance of the transcripts present in WRL68 cells after HuR immunoprecipitation was assessed, and fold differences were plotted. Input, total mRNA in cell extract; unbound, unbound mRNA after immunoprecipitation with HuR antibody; bound, bound mRNA after immunoprecipitation with HuR antibody; and control, bound mRNA after immunoprecipitation with IgG antiboby. (**C–D**) WRL68 and Hep3B cell lines were transfected with miR-16 or In-miR-16 (50 nM). COX-2 and HuR protein levels were analyzed by Western Blot. (**E–F**) WRL68 and Hep3B cell lines were cotransfected with miR-16 (50 nM) and pcDNA3-HuR-GFP expression vector (4 µg). COX-2 and HuR protein levels were analyzed by Western Blot. Data are reported as means±SD of three independent experiments. *p< 0.05 vs. the control condition and # *p< 0.05 vs*. the miR-16 transfection condition.

The miRNAs (miR-16, miR-26b, miR-101, miR-199a, miR-122 and miR-21) were selected by using miRWalk computational analyses, that covers miRNA-targets interactions information produced by 8 established miRNA prediction programs on 3' UTRs of all known genes of Human, Mouse and Rat, i.e., RNA22, miRanda, miRDB, TargetScan, RNAhybrid, PITA, PICTAR, and Diana-microT, and comparing the obtained results with data collected from the literature.

### Western Blot Analysis

Extracts from cells (2–3×10^6^) or from liver tissue were obtained as previously described [32]. For Western blot analysis, whole-cell extracts were boiled for 5 minutes in Laemmli sample buffer, and equal amounts of protein (20–30 µg) were separated by 10–15% SDS-polyacrylamide electrophoresis gel (SDS-PAGE). The relative amounts of each protein were determined with the following polyclonal or monoclonal antibodies: COX-2 (Cayman 160107 and Santa Cruz sc-1747), α-tubulin (Sigma T9026), HuR (Santa Cruz sc-5261), Ago2 (Abcam AB57113) and Caspase-3 (Cell Signaling 9662). After incubation with the corresponding anti-rabbit or anti-mouse horseradish peroxidase conjugated secondary antibody, blots were developed by the ECL protocol (GE Healthcare, Chalfont St Giles, UK). Target protein band densities were normalized with α-tubulin. The blots were revealed, and different exposition times were performed for each blot with a charged coupling device camera in a luminescent image analyzer (Gel-Doc, Bio-Rad) to ensure the linearity of the band intensities. Densitometric analysis was expressed in arbitrary units.

**Figure 6:**
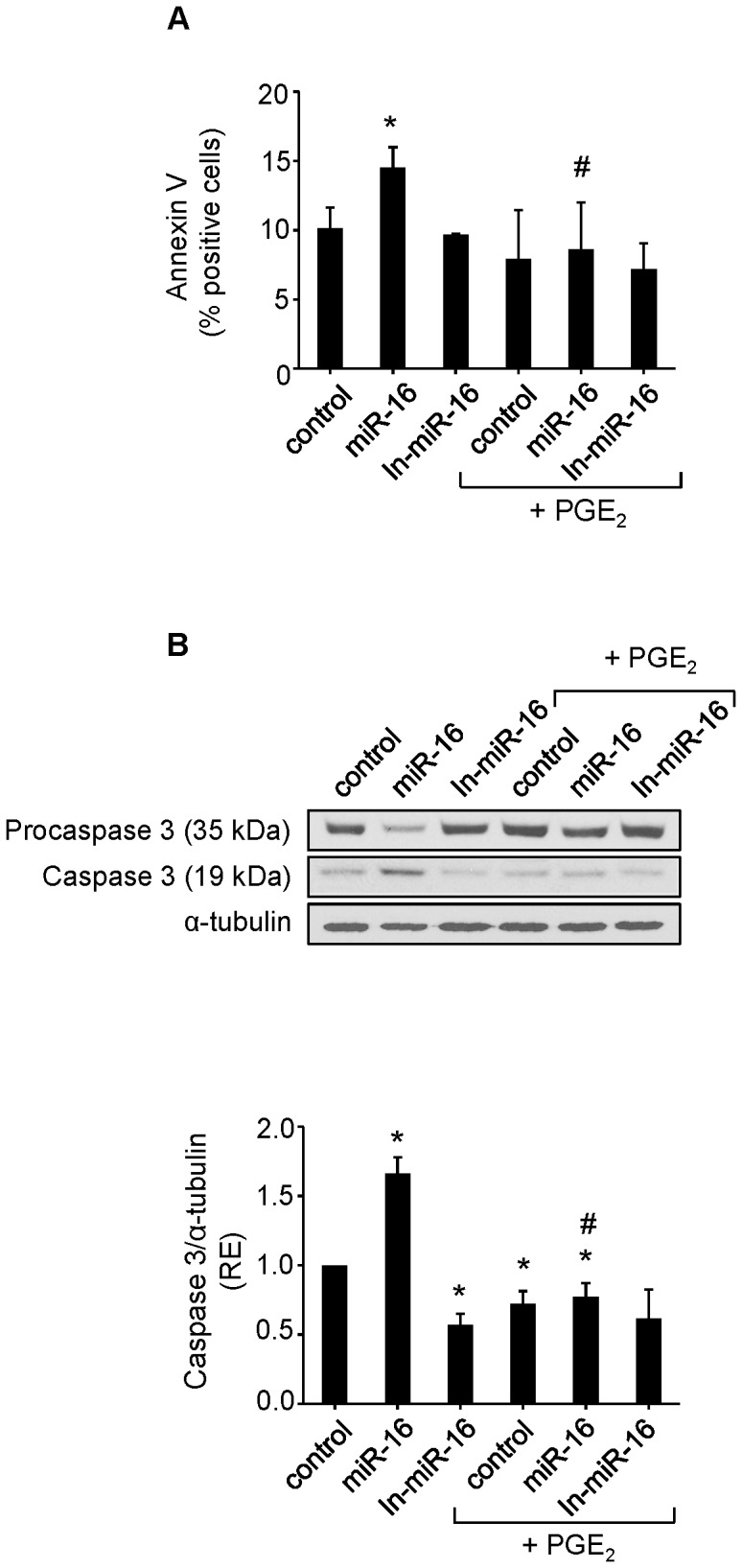
Downregulation of COX-2 by miR-16 increases apoptosis in HCC cells. Hep3B cells were transfected with 50 nM miR-16 or In-miR-16 in the presence or absence of 5 µM PGE_2_ (**A**) Apoptosis was measured with Annexin V-FITC Apoptosis Detection Kit (**B**) Western blot analysis of caspase-3. Results are the means ± SD of three different experiments. *p< 0.05 vs. the corresponding control cells *^#^ p< 0.05 vs.* miR-16 condition.

### Determination of Metabolites

PGE_2_ was determined in culture media by specific immunoassay (Arbor Assays, Ann Arbor, MI, USA). Protein levels were determined with Bradford reagent (Bio-Rad).

### Transfection, Constructs and Luciferase Reporter Assay

The miR-16 precursor (PM10339), which was a double-stranded RNA mimicking the endogenous mature miRNA, the miR-16 inhibitor (In-miR-16, AM10339) which was a single stranded nucleic acid designed to specifically bind to and inhibit endogenous microRNA molecule, their negative controls (miR-NC, AM17110; In-miR-NC, AM17010) and anti-COX-2 siRNA (siCOX-2) (positive control, forward 5′- GGGCUGUCCCUUUACUUCAtt -3′and reverse 5′- UGAAGUAAAGGGACAGCCCtt-3′) were purchased from Ambion (Austin, TX, USA). pPyCAGIP-hCOX-2 was prepared as described previously [33]. Briefly, human COX-2 ORF was amplified by PCR from human full-length COX-2 cDNA cloned into pcDNA1/Amp, and then, was subcloned into *Xho*I-*Not*I restriction site of pPyCAGIP vector. WRL68 and Hep3B cells were seeded in a 6-well plate (3×10^5^cells/well) at 70% confluence. After 24 h, cells were transfected with 50 nM of miR products or 30 nM siCOX-2 using lipofectamine 2000 (Invitrogen, USA) according to the manufactureŕs instructions. After 6 h of incubation at 37°C, transfection medium was replaced with 2 ml of complete medium containing 10% FBS. For the analysis of COX-2 mRNA or protein decay, 5 µg/ml actinomycin-D or 10 µg/ml cycloheximide (Sigma, USA) were added after transfection. Cells were lysated after 48 h for Western blot and RT-PCR analyses.

**Figure 7:**
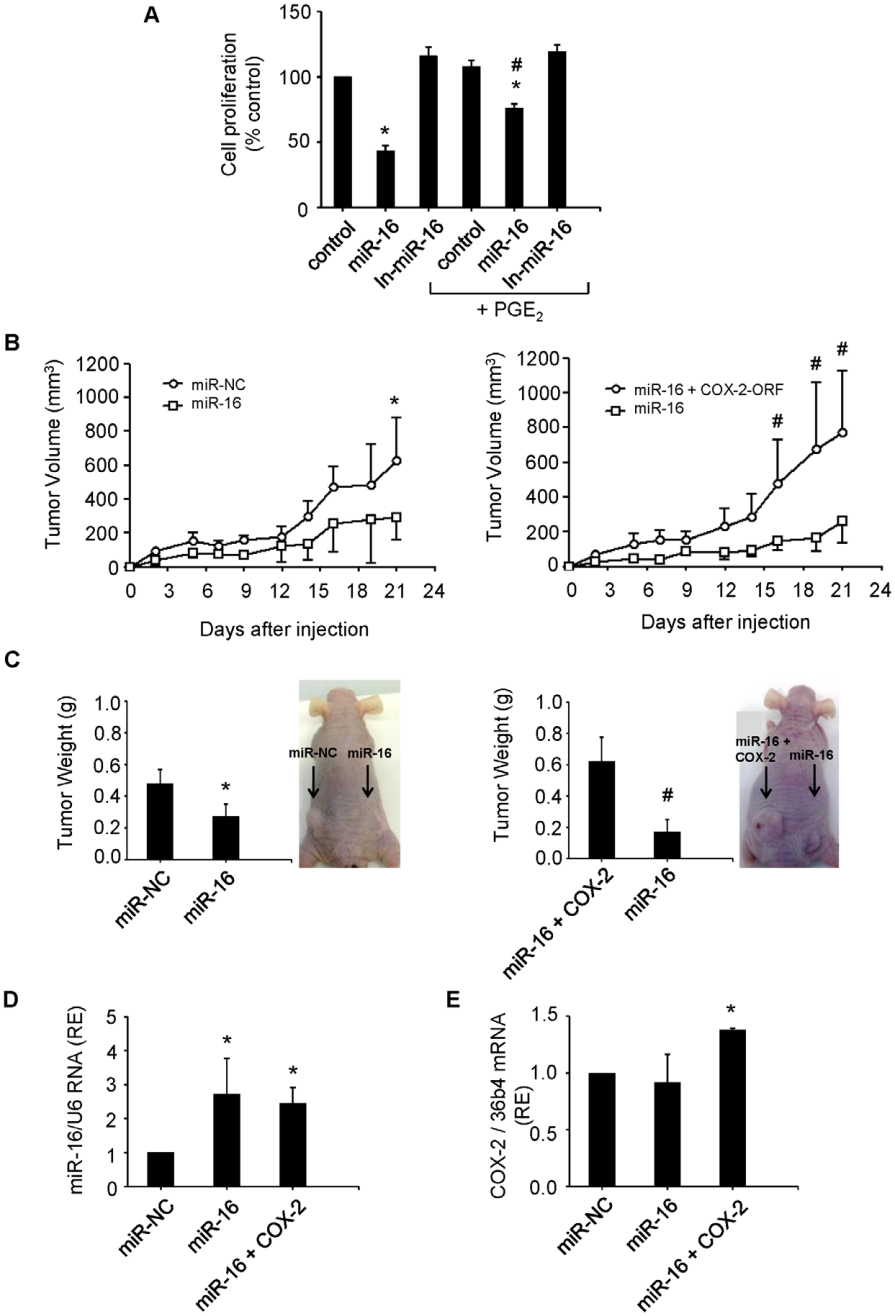
miR-16 suppresses growth of hepatoma cells in vitro and tumorigenicity *in vivo.* (**A**) Hep3B cells were transfected with 50 nM miR-16 or In-miR-16. The effect on cell proliferation was determined by MTT assay at 48 h after transfection. *p< 0.05 *vs*. the control and # *p< 0.05* *vs*. the miR-16 transfection condition. (**B**) Tumor growth curves measured after subcutaneous injection of WRL68 cells transiently transfected with miR-16, miR-NC or miR-16 with a hCOX-2 expression vector lacking 3′ UTR (miR-16+COX-2). Tumor volume (V) was monitored by measuring the length (L) and width (W) with calipers and calculated with the formula (L×W^2^)×0.5. Tumor growth was measured every 2–3 days. *p< 0.05 *vs*. the miR-NC and # p<0.05 *vs.* miR-16 (**C**) Tumor weight and a representative picture of the tumors. At 21 days after injection, mice were killed and tumors were weighed after necropsy. *p< 0.05 *vs*. the miR-NC or # *vs.* the miR-16+ COX-2 condition. (**D–E**) miR-16 and human COX-2 expression in tumors using real-time PCR normalized against U6 RNA levels and refers to miR-NC as 1 or 36b4 mRNA, respectively. *p< 0.05 *vs*. the miR-NC Data are reported as means±SD of three independent experiments or five animals per condition.

To determine whether COX-2 mRNA was located in processing bodies, p-bodies (PB), as a consequence of translational repression, digitonine permeabilization and cellular fractionation of Hep3B cell lines were performed since PB are enriched in the pellet fraction [34]. Hep3B cell lines were transfected with miR-16 or In-miR16 48 hours prior to harvesting at a final concentration of 50 nM. Cells were harvested by trypsinization, washed with cold phosphate buffered saline (PBS) and resuspended in Buffer C [250 mM sucrose, 10 mM Tris-HCl pH 7.5, 25 mM KCl, 5 mM MgCl2, 2 mM DTT, 30 U/ml RNasin, and 0.1% v/v phosphatase-protease inhibitor cocktail (Sigma) containing 50 mg/ml digitonin (Sigma)]. After incubation on ice for 15 min, samples were centrifuged at 1,000 g for 5 min at 4°C. The supernatant was recentrifuged at 14,000 g at 4°C for 5 min and saved as a soluble fraction. The pellet from 1,000 g centrifugation was washed with Buffer C without digitonin and saved as a cell debris pellet. RNA was isolated from each fraction with Trizol reagent and was used for RT-PCR. 5′-3′ exonucleasa (Xrn1) primers; F:GAGAAGCGATTATTGGAAGCCA and R:GCACATTAGGCACTCACTATGTT were used as PB marker.

Using several programs (RNAhybrid, PITA, and RNA22), miR-16 was predicted to associate with the 3′UTR region of COX-2 to different MRE motifs (Table S1). In the present report, miR-16 target site prediction for COX-2 was performed using RNAhybrid [35] and we found one predicted MRE for miR-16 at positions 1195–1217 taking as position 1 the beginning of the 3′ UTR region. The 3′-UTR sequences of COX-2 were retrieved using Ensembl Data base (available: http://www.ensembl.org). Human miRNA sequences were downloaded from the miRBase website (available: http://www.mirbase.org). A fragment of 3′UTR COX-2 mRNA (region 1195–1217, from NM_000963) which include the MRE binding site for miR-16, and a mutant variant were cloned into pGL3-Promoter vector (pGL3-empty, Promega, USA) downstream firefly luciferase gene (*Sac*I, *Hind*III sites) to obtain the luciferase reporter constructs (pGL3-seed and pGL3-mut, respectively). Sequences cloned: 5′-ctttatctcagtcttgaagccaattcagtaggtgcattggaatcaagcctga-3′ (seed); 5′-ctttatctcagtcttgaataaccttcaggagggtaattggaatcaagcctga-3′ (mut).

A DNA fragment containing 2.5 kb corresponding to the full length 3′UTR region of the human COX-2 gene was amplified by PCR and cloned into the pGEM-T easy vector (Promega) to construct pGEMCOX-2/3′UTR. The fragment was obtained by XbaI/BamHI digestion and subcloned in the pGL3-Promoter vector (pGL3-empty, Promega) to construct the pGL3-UTR vector. Mutagenesis was performed by means of the QuickChange Site-Directed Mutagenesis Kit (Stratagene, La Jolla, CA. USA) using pGEMCOX-2/3′UTR as a template. All the constructions were confirmed by nucleotide sequencing.

Cells (3×10^4^ cells/well) were seeded in 24-wells plate and transfected for 6–12 h with pGL3-empty (750 ng), pGL3-seed (750 ng), pGL3-mut (750 ng), pGL3-UTR (750 ng), pGL3-UTR mut (750 ng), pRL-SV40 vector (50 ng, Promega, USA), miR-16 (50 nM), In-miR-16 (50 nM) or miR-NC (50 nM) or a different combinations of them using lipofectamine 2000 reagent protocol. Cells were harvested 48 h after transfection and cell lysates were used for Dual-Luciferase® Reporter Assay System analysis, according to the manufacturer’s instructions (Promega, USA). HuR expression vector, pcDNA3-HuR-GFP, was kindly provided by Dr.M. Gorospe (Laboratory of Molecular Biology and Immunology, NIA-IRP, NIH, Baltimore, USA). When transfection was performed with pcDNA3-HuR-GFP (4 µg) and pPyCAGIP-hCOX-2 or control vector (4 µg) cells were seeded in 6-wells plate.

### Immunoprecipitation and PCR Analysis

The binding of miR-16 to COX-2 mRNA, HuR to COX-2 mRNA and the binding of HuR to miR-16 were analyzed by immunoprecipitation and PCR analysis. The immunoprecipitation was carried out in the lysis buffer (10 mM Tris/HCl, pH 8, 150 mM NaCl, 1% NP40, 0.1% azide, and protease inhibitor cocktail). Total crude extracts (500 µg) from WRL68 and Hep3B cells were immunoprecipitated with 10 µg Ago2, HuR, or IgG antibody and mixed 2 hours at 4°C. An equal volume of protein A/G sepharose was added per immunoprecipitation and mixed overnight at 4°C. The protein/G sepharose was pelleted at 1500 rpm for 2 minutes at 4°C. For the elution of bound RNA, beads were resuspended in the lysis buffer described above, supplemented with 10 µg tRNA from *Escherichia coli* and 80 µg of proteinase K. The mixture was incubated at 50°C for 45 minutes. The RNA was purified using TRIzol reagent (Invitrogen, Carlsbad, CA), reverse transcriptased and PCR amplified with COX-2, miR-16, U6 or actin primers. The PCR reaction was performed at 95°C for 5 min, followed by 30 cycles of 95°C for 30 s, 52°C for 30 s, and 72°C for 1 min, with the following primers; hCOX-2 forward 5′- ATCTACCCTCCTCAAGTCCC-3′and reverse 5′- TACCAGAAGGGCAGGATACAG-3′, actin forward 5′- GCTCACGGAGGCACCCCTGAA -3′and reverse 5′- CTGATAGGACATTGTTAGCAT -3′, miR-16 forward 5′- TAGCAGCACGTAAATATTGGCG -3′and the universal reverse primer provided in the NCode™ miRNA first-strand cDNA synthesis kit (Invitrogen), U6 snRNA forward 5′- CTTCGGCAGCACATATACT -3′and reverse 5′- AAAATATGGAACGCTTCACG -3′. PCR products were visualized by electrophoresis in SYBR Safe DNA gel stain (Invitrogen) agarose gels.

### Analysis of Cell Proliferation

Cell proliferation was determined by the MTT (3-[(4, 5-dimethylthiazol-2-yl)-2, 5′diphenyltetrazolium bromide]) assay (Sigma). Cells (8×10^3^) were seeded on 96-well plates in DMEM supplemented with 10% FBS. After transfection with different conditions, cells were treated with 20 µl of MTT solution (2 mg/ml) for 4 h at 37°C. The medium was removed and DMSO was added to dissolve the blue formazan residue. The optical density was measured at 570 nm.

### Evaluation of Apoptosis

Apoptosis was detected by flow cytometry using Annexin V-FITC Apoptosis Detection Kit (BD Pharmingen, San Diego, CA, USA). Briefly, cells were collected and washed in cold PBS. After centrifugation at 4°C for 5 min at 1000 rpm, cells were double stained with Annexin V-FITC and PI for 15 min at room temperature in the dark. Early apoptosis is defined by Annexin V^+^/PI^-^ staining and late apoptosis is defined by Annexin-V^+^/PI^+^ staining as determined in a Cytomics FC500.

### Analysis of Tumorigenicity in Nude Mice

Female athymic *nu/nu* mice (6 weeks old) were obtained from Charles River Laboratories (Wilmington, MA). All the experiments were performed in accordance with the animal care guidelines of the European Union (2010/63/EU), and approved by the Bioethical Committee from Consejo Superior de Investigaciones Científicas (reference project SAF2010/16037). The animals were kept under pathogen-free conditions and were given an autoclaved standard diet and water *ad libitum* and treated according to the Institutional Care Instructions (Bioethical Commission from Consejo Superior de Investigaciones Científicas, CSIC, Spain). WRL68 cells were transfected *in vitro* with 50 nM miR-NC or miR-16 and pPyCAGIP-hCOX-2 ORF (hCOX-2 expression vector lacking COX-2 3′ UTR) by using lipofectamine 2000. At 24 h after transfection, 5×10^6^ viable cells suspended in PBS were injected subcutaneously into both flanks of the *nu/nu* mice (5 mice per group). Tumor growth was measured every 2 or 3 days. At 21 days after injection, mice were killed and tumors were weighed after necropsy. Tumor volume (V) was monitored by measuring the length (L) and width (W) with calipers and calculated with the formula (L×W^2^)×0.5.

### Data Analysis

Data are expressed as mean ± S.D. (n ranged from three to five independent experiments). Statistical significance was estimated with the Student’s two-tailed *t* test for unpaired observations, Spearman r test for nonparametric correlations and the Mann-Whitney U test was used for ordinal variables using the statistical software GraphPad Prism 5. A *p* value < 0.05 was considered significant.

## Results

### miR-16 and COX-2 Correlate Inversely in Hepatoma Cell Lines

To examine whether COX-2 expression is under the control of miRNAs, we determined the expression pattern of COX-2 and selected miRNAs in four hepatoma (HCC) cell lines (HepG2, PLC/PRF/5, HuH-7, Hep3B) and in a cell line derived from human liver embryo (WRL68), using human hepatocytes (HH) as control (Fig. 1A). We found that each cell line expresses different levels of COX-2 protein and mRNA. WRL68 exhibited the highest COX-2 mRNA levels whereas HepG2 and PLC/PRF/5, two differentiated liver carcinomas, showed low levels of COX-2 mRNA and protein (Fig. 1A). The expression profile of six miRNAs (miR-16, miR-26b, miR-101, miR-199a, miR-122 and miR-21) was analyzed in HCC cell lines (Table 1). In almost all HCC lines analyzed, miR-16 expression was lower than in control hepatocytes (HH), whereas COX-2 protein levels were higher (Fig. 1A). We decided to compare the COX-2 protein/mRNA ratio (as an index of translational inhibition) of the cell lines with the selected miRNAs levels. Among the six miRNAs analyzed, the expression of miR-16 showed the highest inverse correlation with the COX-2 protein/mRNA ratio (R^2^ = 0.858, p = 0.016) (Fig. 1B), suggesting that miR-16 is involved in COX-2 regulation in hepatoma cell lines.

### miR-16 Regulates COX-2 Expression in HCC Cell Lines

Major approaches to validate miRNA targets use *in vitro* gain-of-function and loss-of-function analyses. We overexpressed miR-16 in HCC cell lines and examined whether it decreases endogenous COX-2 levels. The effect of miR-16 transfection on COX-2 protein expression was evaluated in WRL68 and Hep3B cells and it was compared to one positive control, cells transfected with siCOX-2, and with two different negative controls, cells treated only with lipofectamine and cells transfected with miR-NC. As a further control, the effect of both miR-16 and miR-NC inhibitors were analyzed. WRL68 and Hep3B cells were chosen since they express higher levels of COX-2 protein. miR-16 caused a decrease in COX-2 protein levels within 48 h of transfection in both cell lines (Fig. 2A–B). Moreover, the transfection of In-miR-16 induced an increase of COX-2 protein mainly in Hep3B cells. COX-2 mRNA levels were also evaluated and no significant changes was observed following the different treatments with the exception of siCOX-2 transfection (Fig. 2A–B). These results provide further evidence that COX-2 mRNA is post-transcriptionally controlled by miR-16. Released PGE_2_ levels are in good agreement with COX-2 protein changes (Fig. 2C–D).

### miR-16 Binds COX-2 mRNA and Inhibits its Translation

To establish whether the effect of miR-16 on COX-2 expression was mediated through a direct miRNA:mRNA interaction, we performed a RNA immunoprecipitation (RNA-IP) assay in WRL68 cells transfected with miR-16. Immunoprecipitation of total lysates was carried out with an antibody against Argonaute 2 (Ago2), a major component of the microRNA associated to multiprotein RNA-induced-silencing complex (RISC) [36]. As shown in Fig. 3A, COX-2 mRNA was present in the Argo2 immunoprecitation samples where miR-16 was expressed whereas capture of the negative control actin mRNA was unchanged. Using several programs (RNAhybrid, PITA, and RNA22), miR-16 was predicted to associate with the 3′UTR region of COX-2 to different MRE motifs (Table S1) and we found one predicted MRE for miR-16 at positions 1195–1217 taking as position 1 the beginning of the 3′ UTR region. To ensure that miR-16 can bind to this predicted region and cause translational repression, we performed a luciferase reporter gene assay in HuH-7 and HepG2 cells with low levels of miR-16. We cloned the 3′UTR region of COX-2 containing the miR-16 putative binding site (seed region) and a mutant variant downstream the Luc gene in pGL3-vector (pGL3-seed and pGL3-mut, respectively) (Fig. 3B). The luciferase activity significantly decreased after cotransfection with both pGL3-seed and miR-16, when compared to positive control (cells transfected only with pGL3-seed). The transfection of In-miR-16 increased the luciferase activity while the transfection of miR-NC had no effects. Moreover, we did not observe variations of the luciferase activity in cells cotransfected with pGL3-mut and miR-16, in comparison to cells transfected only with pGL3-mut (Fig. 3C–D). The results suggest that miR-16 could specifically bind to the 3′UTR region of COX-2 and represses COX-2 translation reinforcing the hypothesis that COX-2 mRNA is a direct target for miR-16. The effect was similar using 3′ UTR full length region of COX-2 (Figure S1).

To further support the hypothesis that miR-16 is involved in the down-regulation of COX-2 translation, we tested the expression of COX-2 in Hep3B cells after transfection with siCOX-2 or miR-16, in the presence of the transcription inhibitor actinomycin-D. We found a decrease of COX-2 protein in both cases (Fig. 4A–B). However, siCOX-2 induced a rapid decay of COX-2 mRNA (t_1/2_ = 3 h) while the transfection of miR-16 did not show significant mRNA decay when compared to negative controls (cells treated only with lipofectamine and cells transfected with miR-NC; t_1/2_ = 7 to 9 h) (Fig. 4C). We performed a similar experiment in the presence of the protein synthesis inhibitor, cycloheximide (CHX) and the results obtained reveal that both miR-16 and CHX induced a rapid decay of COX-2 protein with a synergistic effect (Fig. 4D–E). Furthermore, when Hep3B cells were treated with digitonin and fractionated after transfection with miR-16 in order to localize COX-2 mRNA in soluble or P-bodies (PB) fractions [34], the amount of COX-2 mRNA present in PB was more than 90%, suggesting inhibition of translation. Instead, in Hep3B cells transfected with lipofectamine, COX-2 mRNA is present in the soluble fraction, where polysomes are located. A similar distribution of COX-2 mRNA was observed upon transfection of Hep3B cells with In-miR-16 (Fig. 4F–G). The results demonstrate that miR-16 interacts with COX-2 mRNA and promotes COX-2 protein decrease mostly through a translational repression mechanism.

### HuR Antagonizes miR-16 Activity in Regulating COX-2 Expression in Hepatoma Cell Lines

It is well known that HuR and other RNA-binding proteins bind to and regulate COX-2 expression and determine the fate of COX-2 translation [37], [38]. However, a recent work [39] has demonstrated that miR-16 inversely correlates with HuR protein levels in human breast carcinoma. RNA immunoprecipitation (RNA-IP) was performed to determine whether HuR would associate with COX-2 and whether there is a direct interaction between HuR and miR-16 in WRL68 cell line. As shown in Fig. 5A, COX-2 mRNA was present in the HuR immunoprecipitates, whereas capture of the negative control actin mRNA was unchanged. Moreover, when RNA-IP was performed, miR-16 was also present in the HuR immunoprecipitates (Fig. 5B). To study the relationship between miR-16 and HuR in HCC cell lines, we determined whether HuR levels were altered by miR-16 transfection. As shown in Fig. 5C–D, overexpression of miR-16 in WRL68 and Hep3B cell lines led to a substantial decrease in HuR protein levels. To determine whether miR-16-mediated COX-2 protein loss was due in part to a decrease in HuR expression, Hep3B and WRL68 cell lines were cotransfected with miR-16 and HuR expression vectors. As shown in Fig. 5E–F, miR-16 inhibited the COX-2 and HuR protein levels in both cellular types; however, in the presence of HuR, the ability of miR-16 to downregulate COX-2 protein levels was partially abolished.

### miR-16 Down Regulation of COX-2 Sensitizes HCC Cells to Apoptosis

To further establish a functional relationship between miR-16 and COX-2, we tested whether COX-2 expression was required to miR-16-dependent induction of apoptosis. Overexpression of miR-16 promoted apoptosis in Hep3B hepatoma cells. However, the effect of miR-16 on apoptosis was partially attenuated by treatment of cells with PGE_2_ (Fig. 6A). Western blot analysis of active caspase-3 showed an increase in the pro-apoptotic protein by the effect of miR-16 and this effect was also reverted in the presence of PGE_2_ (Fig. 6B). These results suggest that miR-16 may exert its pro-apoptotic function partially through decreasing COX-2 expression.

### mir-16 Suppresses the Growth of Hepatoma Cells in vitro and *in vivo*

We sought to determine whether miR-16 affects the growth of hepatoma cell lines assessed by the MTT reduction assay. As indicate in Fig. 7A, the growth of Hep3B cells transfected with miR-16 was significantly decreased relative to control cells. Transfection of the cells with miR-16 decreased cell growth up to 40%, being restored to 70% in the presence of PGE_2_. To further analyze the effect of miR-16 on hepatoma cell growth *in vivo*, the WRL68 cells were transiently transfected with miR-16, miR-NC or miR-16 together with a human COX-2 expression vector that lacks the 3′ UTR and, therefore, it cannot be regulated by miR-16. Then the transfected cells were subcutaneously injected into athymic *nu/nu* mice. The mice were followed by the observation of xenograft growth for 3 weeks. We found that miR-16 led to a significant reduction in the volume and weight of the tumor comparing with the mice injected with miR-NC. COX-2-dependent production of PGE_2_ increased the volume and the weight of tumors comparing with miR-16 (Fig. 7B–C). The expression of intratumoral miR-16, measured by real-time PCR, increased in tumors injected with cells transfected with miR-16 compared with miR-NC without being modified by COX-2 overexpression (Fig. 7D). Moreover, human COX-2 expression was detected in the tumors 21 days after the injection (Fig. 7E). These results agree with the *in* *vitro* data (Fig. 7A) and suggest that miR-16 inhibits the proliferation of hepatoma cells, among other mechanisms, through downregulation of COX-2.

### Inverse Correlation between miR-16 and COX-2 Expression is Observed in HCC Human Biopsies

Since miR-16 regulates COX-2 expression by binding to the MRE in the 3′-UTR COX-2 and by inhibition of HuR in HCC cell lines, we evaluated the relationship between miR-16, HuR and COX-2 mRNA/protein expression in individual tumoral (T) and paired non-tumoral (NT) HCC human samples. COX-2 mRNA and protein were higher in NT tissue compared to T counterparts, like HuR protein and mRNA (Figure S2A-C) whereas miR-16 levels in HCC tissues tended to be higher in T than in NT tissue (Figure S2D) and inversely correlated with COX-2 protein levels (Figure S2E).

## Discussion

In this study we have analyzed whether COX-2 could be regulated by miRNAs or RNA-binding proteins in human hepatoma cell lines and human HCC specimens and whether COX-2 levels in human HCC correlate with an altered expression of these miRNAs. Our results show that miR-16 directly silences COX-2 expression in hepatoma cells and indirectly through the downregulation of HuR. Moreover, a reduced miR-16 expression correlates with high levels of COX-2 in liver from HCC patients. From a functional point of view, COX-2 down-regulation by miR-16 increased apoptosis and decreased cell proliferation in human hepatoma cell lines.

Several lines of evidence suggest that COX-2 signaling is implicated in hepatocarcinogenesis and that COX-2 inhibitors prevent HCC cell growth *in vitro* and in animal models [40]. Increased COX-2 expression has been found in human HCC; however, although COX-2 expression is elevated in the early stages of HCC, many questions remain unsolved regarding the sufficiency of COX-2 to induce/contribute to tumorigenesis. The mechanisms regulating the expression of COX-2 at specific stages of HCC development remain unknown. Moreover, recent data and our present results indicate that COX-2 mRNA levels are significantly higher in the adjacent liver than in the HCC and lower in HCC than in nonalcoholic steatohepatitis [12]. Our previous work demonstrated that COX-2 expression is not sufficient to enhance malignant transformation induced by dyethylnitrosamine in a model of transgenic mice expressing COX-2 in hepatocytes [33]. These results suggest that COX-2 could be related to the inflammatory response occurring in the early phases of chronic liver disease and eventually contribute to the induction of hepatocarcinogenesis.

Several reports describe COX-2 overexpression as a critical step contributing to various facets of colon cancer and growing evidence indicates that this overexpression is facilitated through loss of ARE-mediated mRNA decay [41]. In CRC cells, a variant of COX-2 mRNA lacking the distal region of the 3′UTR was stabilized upon cell growth to confluence [28]. These findings suggest that COX-2 mRNA can escape rapid decay through the use of alternative polyadenylation sites, resulting in deletion of potential 3′UTR regulatory elements. This phenomenon appears to be common in colon cancer cells [42]. Moreover, it has been described a common polymorphism in the 3′UTR of COX-2 and this variant is associated with lung cancer risk [43]. Nevertheless, no data are available concerning the loss of ARE-mediated post-transcriptional regulation of COX-2 or even polymorphisms in the ARE elements in HCC.

There are several miRNAs abundantly expressed in adult liver tissue [44], [45] and the liver displays a differential miRNA expression profile in HCC. Microarray analysis showed altered expression of some miRNAs in hepatomas such as let-7a, miR-21, miR-23, miR-130, whereas the hepato-specific miR-122a and others were found downregulated in 70% of HCCs and in HCC-derived cell lines [20], [46], [47], as reported in our data (Table 1). Murakami et al. [48] showed a correlation between miR-222, miR-106a, miR-92, miR-17-5p, miR-20 and miR-18 and the degree of differentiation suggesting an involvement of specific miRNAs in the progression of the disease. However, the molecular mechanisms underlying transcriptional regulation of miRNA genes in the liver remain poorly established and different transcription factors, such as hepatocyte nuclear factor 1α, c-myc, STAT-3 and NF-κB have been implicated [25], [49]. Understanding the contribution of desregulated miRNAs to HCC requires the identification of gene targets and in this sense, cyclin G1 and the PTEN tumor suppressor gene have been found to be regulated by miR-122a and miR-21, respectively [50], [51].

The 3′-UTR of COX-2 is complex and contains multiple copies of AREs and MREs which, when bound to specific ARE-binding factors or miRNAs, influence COX-2 stability and translational efficiency [17]. Work investigating the role of COX-2 during embryo implantation identified the miRNAs, miR-101a and miR-199a as regulators of COX-2 [22], [23]. miR-101a also controls mammary gland development by regulating COX-2 expression [52]. In the context of colon cancer cell lines and colon tumors, miR-101 inhibited COX-2 translation [24]. Young et al. [36] demonstrated that miR-16 binds the COX-2 3′UTR and inhibits COX-2 expression by promoting mRNA decay in colon cancer. However, the functional consequences of miR-16 associated with HCC progression have not been established. The present results demonstrate that miR-16 regulates COX-2 expression in HCC cells by binding directly to the MRE response element in the COX-2 3′UTR and this binding inhibits mainly COX-2 translation without affecting significantly mRNA decay. It has been described by Huang et al. that miR-16 decreased the association of its target mRNA with polysomes in 293T and HeLa cells by mediating the association of mRNA with processing bodies (P-bodies), since localization of mRNAs to these structures is a consequence of translational repression [34]. A similar assay has been used previously to support the PB-to-cytosol relocalization of mRNAs relieved from miRNA repression by treatment with antisense oligonucleotides [53]. Our results clearly show that COX-2 mRNA was located in P-bodies (>90%) after transfection with miR-16, inhibiting its translation.

Next, we investigated the effect of COX-2-mediated inhibition by miR-16 in hepatocarcinogenesis. Our data show that the ectopic expression of miR-16 repressed cell proliferation of hepatoma cells *in vitro* and tumor growth *in vivo*, and these effects were partially reverted by treatment with PGE_2_. Furthermore, COX-2 inhibition mediated by miR-16 promoted apoptosis in HCC cells by increasing apoptotic proteins such as caspase-3.

Various cytoplasmic proteins have been observed to bind to the COX-2 ARE. To date, 16 different RNA-binding proteins bind the COX-2 3′UTR promoting mRNA decay, mRNA stabilization or translational silencing [41]. The HuR protein is a ubiquitously expressed member of the ELAV (Embryonic-Lethal Abnormal Vision in Drosophila) family of RNA-binding proteins. HuR contains three RNA recognition motifs with a high affinity and specificity for AREs and its overexpression stabilizes transcripts and promotes their translation [54]. HuR is localized predominantly in the nucleus and the ability of HuR to promote mRNA stabilization requires its translocation to the cytoplasm. Different cellular signals known to activate MAPK pathways, the PI-3 kinase pathway and the Wnt signaling pathway have been shown to trigger cytoplasmic HuR localization [55]. HuR has been identified as a trans-acting factor that promotes COX-expression and it is known that cytoplasmic HuR expression correlates with poor clinical outcome and with COX-2 expression in ovarian carcinoma [29], human keratinocytes after UVB irradiation [56] and in colon carcinogenesis [37], [57]. It is known that HuR is overexpressed in CRC cells and tumors, where elevated HuR levels can impede ARE-mediated decay [37]. However, the expression of HuR in HCC is not reported. HuR binds to COX-2 and increases/maintains COX-2 expression in HCC cells. Moreover, miR-16 is also present in the HuR immunoprecipitated and the analysis of miR-16 predicted target genes determined by using the algorithms miRWalk showed that among miR-16 target genes one is HuR. miR-16 interacts with HuR mRNA in the 3′UTR and represses HuR translation in human breast cancer cells [39]. Indeed, Dixon et al. [37] reported a direct interaction between HuR and miR-16 promoting the downregulation of miR-16 and targeting COX-2 in colon cancer cells. Our data are in agreement with the proposed interaction between miR-16 and HuR mRNA in HCC cells and suggest two different mechanisms for miR-16 to inhibit COX-2: by binding directly to the MRE response element in the COX-2 3′-UTR and by decreasing the levels of HuR through a direct interaction. Our results show HuR expression, protein and mRNA, in both NT and T tissue from HCC biopsies, paralleling COX-2 expression. Moreover a reduced miR-16 expression tends to correlate to high levels of COX-2 protein in liver from patients affected by HCC. Therefore, the reduced expression of miR-16 in those HCC with a high COX-2 expression may contribute to the promotion of cell proliferation and the inhibition of apoptosis and consequently facilitate the development of these types of tumors. Our data suggest an important role for miR-16 in HCC and implicate the potential therapeutic application of miR-16 in those HCC with a high COX-2 expression.

## Supporting Information

Figure S1 miR-16 downregulates COX-2 by binding its 3′UTR. A luciferase assay was carried out on HuH-7 cell line using pGL3-UTR reporter vectors. Firefly luciferase activity was evaluated 48 h after co-transfection with pGL3-empty, pGL3-UTR or pGL3-UTR mut (750 ng) and miR-16 (50 mM). Data were normalized against renilla luciferase activity (all samples were co-transfected with 50 ng pRL vector and refer to the positive control, pGL3 empty vector). Data are reported as means ± SD of three independent experiments. *p<0.05 vs. the pGL3-UTR condition and #p<0.05 vs. the miR-16 transfection condition.
(TIFF)

Figure S2 COX-2 correlates inversely with miR-16 and directly with HuR in HCC human biopsies. (**A**) COX-2 and HuR protein expression were analyzed in both tumor (T) and their paired non tumor (NT) tissues by Western Blot in a total of 7 pairs of matched tissue specimens. Corresponding densitometry analysis is shown and the relative expression of each sample is refer to that in one non tumor tissue sample NT. (**B–D**) The expression of COX-2 mRNA, HuR mRNA and miR-16 were analyzed using real-time PCR in NT and T tissue. *p< 0.05 vs. NT samples (**E**) COX-2 protein levels were compared to miR-16 expression in T samples. Data were normalized against α-tubulin and U6 RNA levels, respectively.
(TIFF)

Table S1 Several binding sites for miR-16 wihtin COX-2 3′UTR, predicted by diferent algorithms. Using several programs (RHAhybrid, RNA22, PITA, targeScan, microRNA.org), miR-16 was predicted to associate with the 3′UTR region of COX-2 to different MRE motifs. The number of binding sites, the positions and the folding energy are indicated for each program. The 3′UTR sequence of human COX-2 was retrieved using Ensembl Data base, and miR-16 sequence for Homo Sapiens was downloaded from mirBase website.
(DOC)
