# Supplementary figures and images for: Cyclooxygenase-2 Is a Target of MicroRNA-16 in Human Hepatoma Cells
Source: PLoS One. 2012 Nov 30;7(11):e50935. doi: 10.1371/journal.pone.0050935 (PMC3511388; doi:10.1371/journal.pone.0050935)

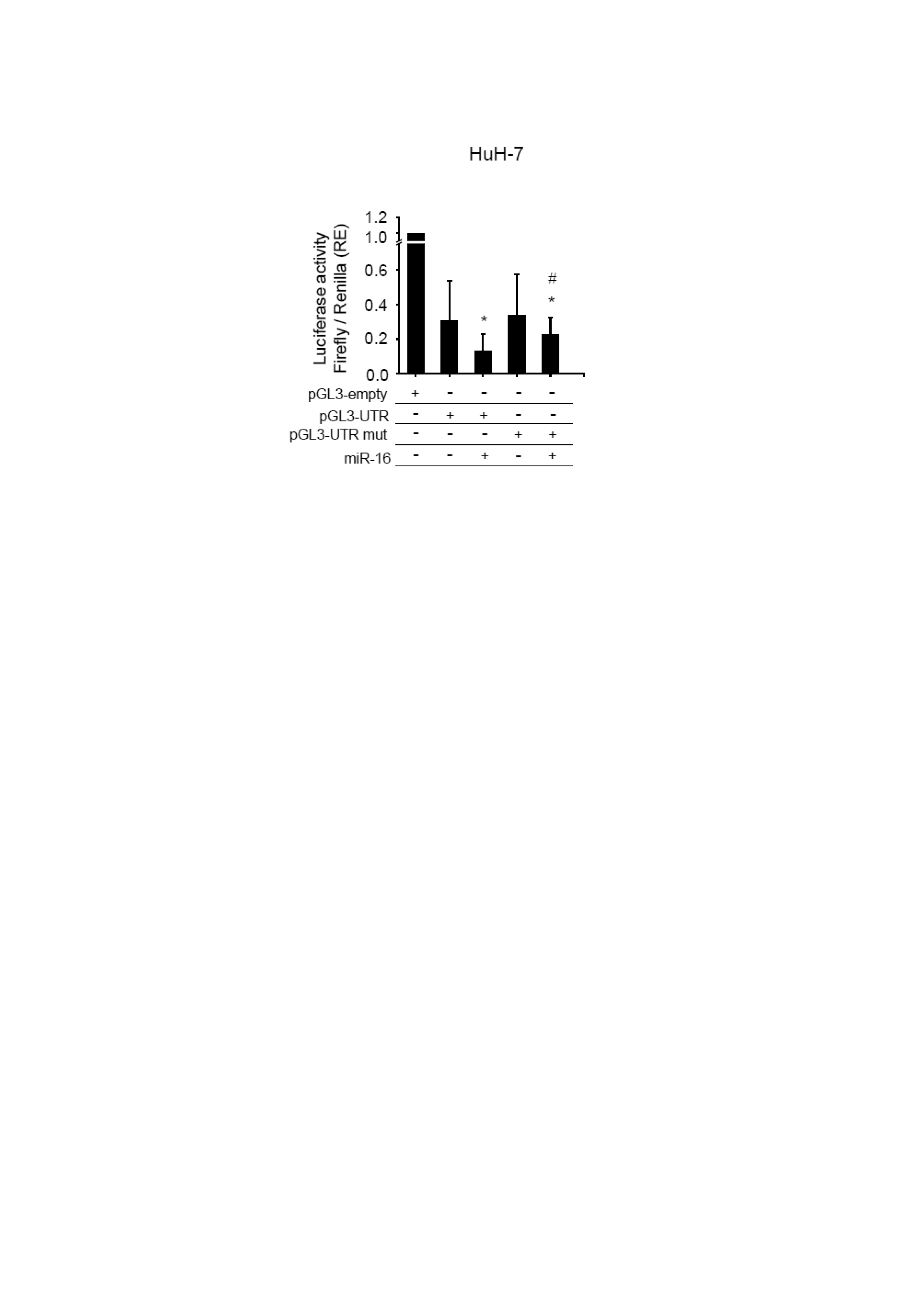

Supplement: Figure S1 — miR-16 downregulates COX-2 by binding its 3′UTR. A luciferase assay was carried out on HuH-7 cell line using pGL3-UTR reporter vectors. Firefly luciferase activity was evaluated 48 h after co-transfection with pGL3-empty, pGL3-UTR or pGL3-UTR mut (750 ng) and miR-16 (50 mM). Data were normalized against renilla luciferase activity (all samples were co-transfected with 50 ng pRL vector and refer to the positive control, pGL3 empty vector). Data are reported as means ± SD of three independent experiments. *p<0.05 vs. the pGL3-UTR condition and #p<0.05 vs. the miR-16 transfection condition. (TIFF) [file pone.0050935.s001.tiff]

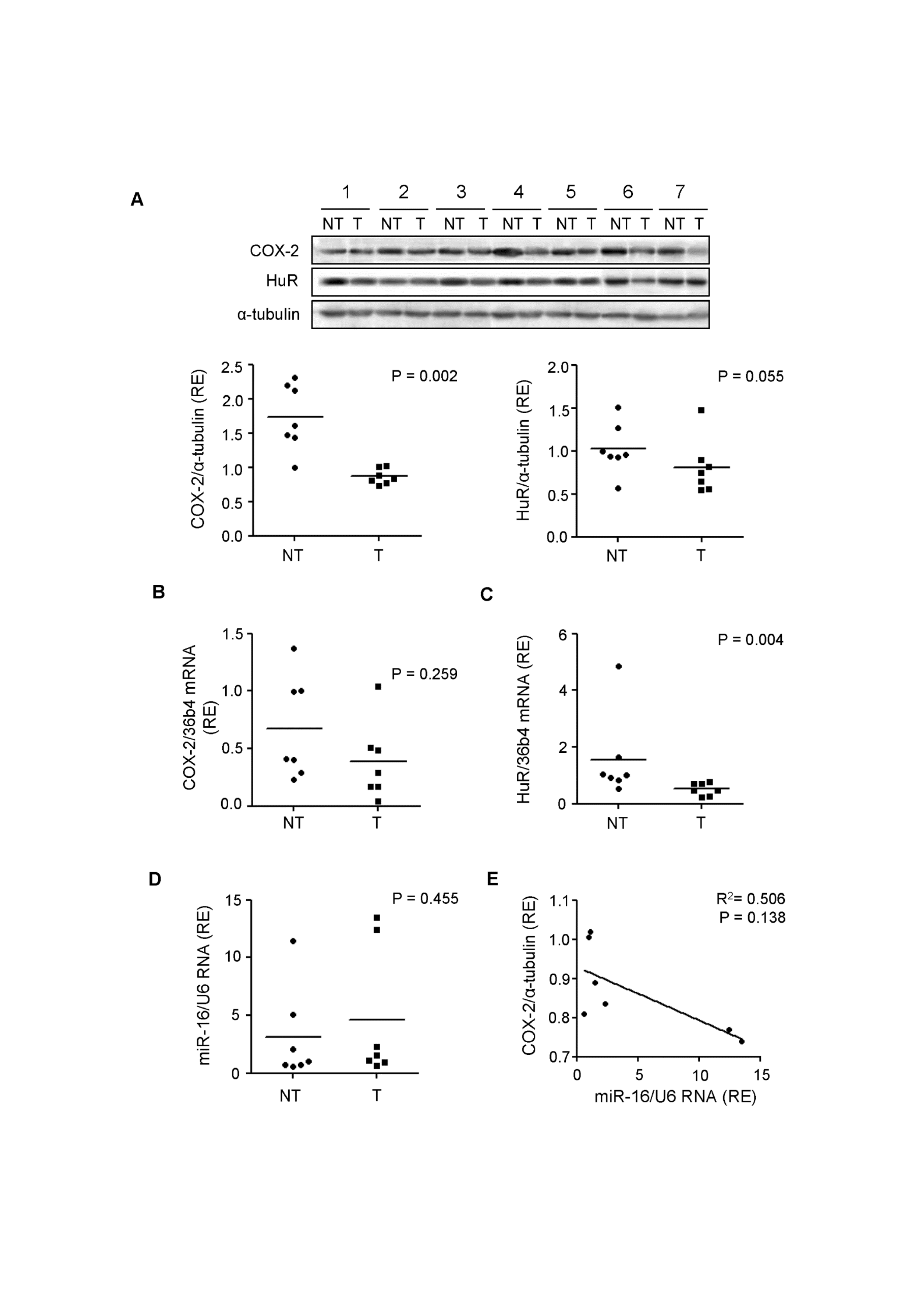

Supplement: Figure S2 — COX-2 correlates inversely with miR-16 and directly with HuR in HCC human biopsies. (A) COX-2 and HuR protein expression were analyzed in both tumor (T) and their paired non tumor (NT) tissues by Western Blot in a total of 7 pairs of matched tissue specimens. Corresponding densitometry analysis is shown and the relative expression of each sample is refer to that in one non tumor tissue sample NT. (B–D) The expression of COX-2 mRNA, HuR mRNA and miR-16 were analyzed using real-time PCR in NT and T tissue. *p< 0.05 vs. NT samples (E) COX-2 protein levels were compared to miR-16 expression in T samples. Data were normalized against α-tubulin and U6 RNA levels, respectively. (TIFF) [file pone.0050935.s002.tiff]
