# Supplementary material for: Cyclooxygenase-2 Is a Target of MicroRNA-16 in Human Hepatoma Cells
Source: PLoS One. 2012 Nov 30;7(11):e50935. doi: 10.1371/journal.pone.0050935 (PMC3511388; doi:10.1371/journal.pone.0050935)
Supplement: Supplementary file 2 [file pone.0050935.s003.doc]

Software	Sites	Position	Folding energy (Kcal/mol)
RNAhybrid	1	1195-1214	-22.5
RNA22	1	169-190	-25.9
PITA	5	179	-11,86
		182	-14.2
		185	-12.9
		702	-9.5
		1238	-15.64
targetScan	0		
microRNA.org	0		
